# Association between interoceptive sensitivity and impaired fasting glucose in early pregnancy: the mediating effect of dietary quality distance and food variety

**DOI:** 10.1186/s41043-026-01418-3

**Published:** 2026-08-11

**Authors:** Xiaohan Tan, Qianqian Tian, Bin Liu, Wei Zhang, Jun Liu, Xiaohua Wang, Omorogieva Ojo, Qing Jiang

**Affiliations:** 1 Division of Endocrinology, the First Affiliated Hospital of Soochow University, Suzhou, 215006 China; 2 School of Nursing, Medical College, Soochow University, Suzhou, 215006 China; 3 Obstetric Department, Affiliated Women’s Hospital of Jiangnan University, Wuxi, 214000 China; 4 Cardiology Division, the First Affiliated Hospital of Soochow University, Suzhou, 215006 China; 5 School of Health Sciences, Faculty of Education, Health and Human Sciences, University of Greenwich, London, SE9 2UG UK

**Keywords:** First trimester, Interoception, Impaired fasting glucose, Dietary quality, Mediation analysis

## Abstract

**Background:**

Early pregnant women often experience changes in their dietary patterns. Interoceptive sensitivity (IS), the ability to detect internal bodily signals, influences appetite regulation and dietary choices, potentially impacting glucose metabolism. Whether IS is a protective factor against elevated FBG in early pregnancy, and poor dietary patterns are linked to impaired fasting glucose (IFG), the relationships between IS, dietary patterns, and IFG remain unclear.

**Methods:**

This cross-sectional study was conducted from December 2024 to June 2025. IS and FBG were recorded in early pregnancy; a three-day dietary diary was applied to record the number of consumed foods and was valued by Chinese Diet Balance Index for Pregnancy (DBI-P), which was used to assess dietary quality distance (DQD) and food variety which comprehensively reflect unbalanced diet and richness of food intake, respectively. Logistic regression analyzed association between IS and IFG. The mediation model test was employed to examine the mediating effect of dietary quality.

**Results:**

A total of 215 patients were included in the study, and the prevalence of IFG was 17.2% (*N* = 37). The scores of IS and its subscale including Emotional Awareness, Self-Regulation, Body Listening, and Trust in IFG group were lower than those in normal group. IS was significantly associated with IFG (OR = 0.491, 95% CI: 0.287, 0.839, *P* = 0.009). The values of DQD especially in Low Bound Score (LBS) were higher in the IFG group, whereas the food diversity was less sufficient than that in the normal group. The DQD and food variety mediated the relationship between IS and IFG, (DQD: indirect = -0.101, 95%CI: -0.241, -0.018; food variety: indirect = -0.074, 95%CI: -0.207, -0.010).

**Conclusion:**

Our findings suggest that IS is significantly associated with impaired fasting glucose in early pregnancy which is mediated by dietary quality, mainly diet quality distance and food variety. The pathway may be that an impaired interoceptive sensitivity drives less optimal dietary choices (high LBS, low food variety), which in turn negatively impacts fasting glucose levels. These findings offer a novel perspective on the potential value of interoception-oriented approaches. Integrating body-awareness training into early pregnancy may represent a promising avenue to improve dietary choices and achieve better glycemic outcomes for pregnant women at risk of metabolic dysfunction.

## Introduction

Gestational diabetes mellitus (GDM) is defined as the onset or first recognition of varying degrees of glucose intolerance during pregnancy, with a global prevalence rate as high as 14.0% [1]. Elevated blood glucose level is an important manifestation of GDM. In early pregnancy, fasting glucose is a powerful predictor for GDM [2]. Impaired fasting glucose (IFG) is a pivotal clinical indicator for identifying women at high risk for GDM [3].

Interoception refers to the process by which the nervous system senses, interprets, and integrates internal bodily signals [4]. It originates from interoceptive signals within the organism [5]. Interoceptive sensitivity (IS) refers to an individual’s tendency and ability to detect internal bodily sensations [5]. The hepatic portal vein in humans contains vagal afferent nerve fibers, which serve as interoceptive tissues. These fibers act as specialized glucose sensors that detect systemic blood glucose fluctuations. Upon detecting these changes, they transmit signals directly to the brainstem and the hypothalamus—the central hubs for appetite regulation and metabolic homeostasis [6]. Importantly, the accurate perception of internal bodily signals may facilitate self-regulatory control over behavior [7]. Individuals with higher IS may be more likely to regulate eating behavior in response to hunger and satiety cues [8]. In other words, they eat only when physiologically hungry and stop promptly upon feeling full, which leads them to preferentially select healthier and more appropriate quantities of food based on their body’s actual needs. Individuals with poor interoceptive ability struggle to differentiate between internal cues (e.g., hunger) and emotional cues, leading to increased eating in response to boredom or negative emotions [9], which may ultimately excessive dietary intake. Thereby, IS may influence whole-body glucose metabolism and associate with glycemic control.

Driven by the surge in hormones like estrogen and progesterone, women in early pregnancy frequently experience a distinct set of dietary symptoms. These can range from nausea and loss of appetite to pronounced cravings and aversions towards specific food categories [10]. As a result, specific food preferences and aversions may promote an unbalanced diet, thereby compromising overall dietary quality and diminishing food variety. These hormones also impact metabolism, leading to IFG and elevated blood glucose levels during pregnancy, especially in early pregnancy. Consequently, pregnant women in their first trimester are more prone to changes in IS and dietary patterns which have subsequently elevated blood glucose levels. However, how IS interacts with dietary quality to influence glycemic control during early pregnancy remains unexplored. We hypothesize that in early pregnancy, IS is associated with impaired fasting blood glucose, which may be mediated by dietary quality.

## Materials and methods

### Study design

This study is a cross-sectional study. Pregnant women who completed prenatal registration and received regular prenatal check-ups at Wuxi Maternal and Child Health Hospital and the First Affiliated Hospital of Soochow University from December 2024 to June 2025 were included in the study. During the first trimester, three professionals distributed paper-based, self-administered questionnaires to the pregnant women. Standardized instructions were provided, and the professionals remained available to answer questions while the participants completed the forms independently. The questionnaires covered the following: [1] demographic and clinical data using general situation questionnaires; [2] the interoceptive sensitivity of pregnant women was evaluated by using the Multidimensional Assessment of Interoceptive Awareness–Version 2 (MAIA-2); [3] the food variety and dietary deviation were evaluated through the Three-Day Diet Diary and the Dietary Balance Index for Pregnancy (DBI-P). The level of physical activity was measured with the International Physical Activity Questionnaire–Short Form (IPAQ-SF); the Generalized Anxiety Disorder 7-item (GAD-7) and the Patient Health Questionnaire-9 (PHQ-9) were used to screen for anxiety and depression symptoms. Meanwhile, the fasting blood glucose levels of the patients in the first trimester were measured.

This study was approved by the Ethics Committee of Soochow University (SUDA20250108H12) and implemented in accordance with the Declaration of Helsinki. All participants signed the informed consent form.

### Participants

The required sample size was determined through a power analysis for binary logistic regression (Z-test family) using G*Power software (version 3.1.9.2; RRID: SCR_013726). Based on an anticipated Odds Ratio (OR) of 0.6, a base event probability (Pr) of 0.18 [11], a two-sided significance level (α) of 0.05, and a statistical power (1 − β) of 80%, the analysis indicated that a minimum sample size of 212 participants was required.

A total of 215 eligible patients were recruited through convenience sampling, and their baseline characteristics were collected. The inclusion criteria were as follows: [1] age ≥ 18 years; [2] singleton pregnancy; [3] provision of informed consent and willingness to participate in the survey. Exclusion criteria included: [1] multiple gestation; [2] miscarriage; [3] stillbirth; [4] history of infectious diseases; [5] pre-existing major medical conditions such as hypertension, diabetes, mental disorders, or cancer; [6] history of allergic conditions, including food allergies.

### Measurements

#### Demographic and clinical data

The questionnaire was independently designed by the research team and mainly included demographic, clinical and obstetric data of pregnant women. Demographic data mainly include age, occupation, educational level, place of residence, personal income, sleep patterns, as well as smoking and drinking status. Clinical data comprised pre-pregnancy body mass index (BMI), alanine aminotransferase levels, past medical history, family history, and medication use. Obstetric data involved gravidity, parity, mode of conception, and symptoms experienced in the first trimester.

#### Interoceptive sensitivity

The Multidimensional Assessment of Interoceptive Awareness–Version 2 (MAIA-2) was employed as a self-report measure to assess subjective interoceptive sensitivity in first-trimester pregnant women. This scale was developed by Mehling et al. in 2018 [12] and translated into Chinese and revised by Teng in 2022 [13]. The Chinese version of the MAIA-2 scale consists of 31 items, which can be divided into 7 subscales: Not-Distracting, Not-Worrying, Attention Regulation, Emotional Awareness, Self-Regulation, Body Listening, and Trust. Responses were collected using a 6-point Likert scale (0 to 5). The overall scale demonstrated good internal consistency, with a Cronbach’s α of 0.822, and subscale α values ranging from 0.656 to 0.838 [13], supporting its reliability and validity for measuring interoceptive sensitivity. In this study, the Cronbach’s α coefficient of this scale was 0.898.

#### Three-Day Diet Diary in the First Trimester

Participants’ food intake was assessed using a three-day dietary record, which included two weekdays and one weekend day. Participants were provided with food weight estimation charts and electronic kitchen scale to aid in accurate assessment of food portions to obtain precise weights. When exact weighing was not feasible, such as when dining out, participants estimated portion sizes using household utensils (e.g., spoons, cups) and photographs of their meals. They were instructed to record the names and estimated weights of all foods consumed over a three-day period.

### Dietary Balance Index for Pregnancy (DBI-P), the food variety and dietary deviation

#### Dietary Balance Index for Pregnancy (DBI-P)

The DBI-P, developed by Li Mengyuan et al. [14] comprises five components with nine individual food categories: grains; vegetables and fruits; dairy products; soybeans and nuts; animal foods (including poultry, aquatic products, and eggs); and food variety (reflected by the number of food types consumed). In this study, dairy products; soybeans and nuts were merged into one component. The intake of grains, fruits, livestock and poultry meat, and eggs is assigned 0 points if it falls within the recommended range. For vegetables, dairy products, soybeans, nuts, and aquatic products, consumption that exceeds the recommended range is still assigned 0 points up to a specified minimum limit. For the DBI-P, one trained professional served as the primary evaluator and performed the main coding of dietary intake. A second professional simultaneously reviewed all coding. Any discrepancies were discussed until consensus was reached. If agreement could not be achieved, a third professional was consulted to make the final decision.

#### The dietary deviation

The DBI-P evaluates dietary deviation using three indicators: the Low Bound Score (LBS), High Bound Score (HBS), and Diet Quality Distance (DQD). The LBS represents the absolute value of the total negative scores across indicators, reflecting the degree of inadequate dietary intake. The HBS denotes the absolute value of the total positive scores, indicating the extent of excessive intake. The DQD is the sum of the absolute values of both positive and negative scores for all indicators, providing a comprehensive measure of overall dietary imbalance. Dietary deviation is stratified based on the DQD score as follows [14]: appropriate (0–12, Q1), low imbalance (13–24, Q2), moderate imbalance (25–36, Q3), and high imbalance (> 36, Q4).

#### The food variety

The food variety was reflected by the number of food types consumed based on Three-Day Diet Diary. This evaluation covers the following 12 food categories: rice and its products, wheat flour and its products, coarse grains and tubers, dark-colored vegetables, light-colored vegetables, fruits, soybeans and nuts, dairy and its products, poultry and its products, red meat and its products, eggs, and fish and shellfish. For the calculation, all foods consumed by pregnant participants in their three daily meals and snacks were recorded, and the three-day average intake for each food category was calculated as the daily intake. A score of 0 is assigned if the intake of a food category meets or exceeds the minimum threshold, and a score of 1 is assigned if it falls below the threshold (the minimum intake for soybeans is set at 5 g, while for all other food categories, it is 25 g). The total score ranges from − 12 to 0 points, where a higher score (closer to 0) indicates better food variety, and a lower score (closer to -12) indicates poorer variety. Participants were then classified into three groups (tertiles) according to their food variety scores: Q1 (< -5.0), Q2 (-5.0 to -4.0), and Q3 (> -4.0).

#### Physical activity

Physical activity levels were assessed using the International Physical Activity Questionnaire–Short Form (IPAQ-SF; RRID: SCR_027364). The questionnaire collects information across four domains: vigorous-intensity activities (such as swimming, running, and climbing stairs), moderate-intensity activities (such as brisk walking, sweeping floors, and cleaning windows), walking (in bouts lasting at least 10 min), and sedentary time (periods longer than 10 min). The IPAQ-SF has been validated in populations aged 18–65 across 12 countries, demonstrating a test–retest reliability correlation coefficient of 0.8 and a criterion validity correlation coefficient of 0.3 [15].

#### Anxiety and depression

Anxiety symptoms were assessed using the Generalized Anxiety Disorder 7-item (GAD-7; RRID: SCR_003666) scale. Participants reported how frequently they were bothered by seven core anxiety symptoms (e.g., feeling nervous or worrying) over the past two weeks [16]. Total scores range from 0 to 21 and are interpreted as follows: 0–4 (non-clinical anxiety), 5–9 (mild), 10–14 (moderate), and ≥ 15 (severe). Depression symptoms were evaluated with the Patient Health Questionnaire-9 (PHQ-9). This instrument includes nine items, each rated by participants on a frequency scale from “not at all” to “nearly every day“ [17]. A total score (0–27) is calculated by summing all items.

#### Blood glucose

Venous blood samples were collected from all participants after an overnight fast of 8–10 h and before breakfast. Fasting plasma glucose (FPG) levels were measured subsequently. In accordance with the American Diabetes Association (ADA) guidelines [3], a fasting blood glucose level of ≥ 5.6mmol/L is defined as impaired fasting blood glucose (IFG), otherwise as normal. This threshold is specifically applied in early pregnancy to identify women with pre-existing or early-onset impaired glucose regulation. Clinically, identifying IFG at this early stage is crucial, as it represents a state of prediabetes and serves as an early warning sign for subsequent metabolic complications later in pregnancy.

### Statistical methods

Statistical analyses were performed using SPSS (Version 29.0; RRID: SCR_002865). Normally distributed continuous data are presented as mean ± standard deviation and were compared using the t-test. Non-normally distributed data are expressed as median with interquartile range and compared using the Mann-Whitney U test. Categorical data are presented as frequency (percentage) and were compared using the Chi-square test or Fisher’s exact test, as appropriate. Variables with an univariate significance of *P* ≤ 0.05 (specifically, pre-pregnancy BMI and education level) were included as independent variables in a binary logistic regression model to explore the association between interoceptive sensitivity and early-pregnancy impaired fasting blood glucose. To examine the relationships between variables, Spearman’s rank correlation analysis was conducted. Subsequently, a mediation model was tested using Mplus (Version 8.11; RRID: SCR_015578) to explore the interrelationships among interoceptive sensitivity, diet quality (assessed by diet quality distance and food variety), and fasting blood glucose levels. The model utilized bootstrapping with 5000 resamples to derive bias-corrected standard errors, and effects were considered statistically significant if the 95% confidence interval did not include zero.

P-value < 0.05 was considered statistically significant.

## Result

Initially, a total of 220 participants were screened for eligibility. After excluding 5 individuals with incomplete data (3 failed to complete the MAIA-2 questionnaire, and 2 provided insufficient dietary intake records), 215 were included in the final analysis. According to the criteria for IFG [3], these participants were categorized into a normal group (*n* = 178) and an IFG group (*n* = 37).

### The Status of interoceptive sensitivity in women with early pregnancy

The quartiles of the participants’ IS scores in early pregnancy were 2.7(2.0,3.2) points. The specific scores of each dimension were shown in Table 1. The comparison results between groups indicated that the total score of IS and the scores of dimensions in emotional awareness, self-regulation, body listening and trust) in the IFG group were significantly lower than those in the normal group (*P* < 0.05).

**Table 1:** Comparison of interoceptive sensitivity levels in group with early pregnancy

Variables		Total (*N* = 215)	Normal group	IFG group	Z	*P*
			< 5.6 mmol/L (*N* = 178)	≥ 5.6 mmol/L (*N* = 37)		
Total score		2.7(2.0,3.2)	2.7(2.10,3.26)	2.1(1.8,2.7)	-3.284	0.001^**^
Sub- scales	Not-Distracting	3.5(2.3,4.3)	3.5(2.3,4.3)	3.3(2.5,4.7)	-0.025	0.980
	Not-Worrying	2.6(2.0,3.0)	2.6(2.0,3.0)	2.6(2.2,3.0)	-0.613	0.540
	Attention Regulation	2.3(1.1,3.1)	2.4(1.1,3.3)	2.0(1.0,2.7)	-1.801	0.072
	Emotional Awareness	2.8(1.6,3.8)	3.0(2.0,3.8)	2.4(0.6,3.2)	-2.459	0.014^*^
	Self-Regulation	2.5(1.3,3.5)	2.8(1.5,3.5)	1.8(1.0,2.5)	-3.455	0.001^**^
	Body Listening	2.3(1.0,3.0)	2.3(1.0,3.0)	1.3(0.3,3.0)	-2.081	0.037^*^
	Trust	3.0(1.3,4.0)	3.0(2.0,4.0)	2.3(1.0,3.0)	-3.051	0.002^**^

IFG: Impaired fasting blood glucose; Z: Mann-Whitney U test; ^**^: *P* < 0.01; ^*^: *P* < 0.05

### Demographic, clinical and obstetric data in early pregnancy

In this study, the average age of the participants was 30.6 ± 4.3 years old. Compared with the normal group, participants in the IFG group showed lower educational attainment and a higher pre-pregnancy BMI. See Table 2 for details.

**Table 2:** Demographic, clinical and obstetric data between normal group and IFG group in early pregnancy

Variables		Total (*N* = 215)	Normal group	IFG group	T/χ^2^/Z	*P*
			< 5.6 mmol/L (*N* = 178)	≥ 5.6 mmol/L (*N* = 37)		
		x̄ ± s/M (P25,P75)/*n*(%)	x̄± s/M (P25,P75)/*n*(%)	x̄± s/M (P25,P75)/*n*(%)		
**Demographic data**						
Age (years)		30.6 ± 4.3	30.0 ± 4.1	29.0 ± 3.4	-0.758	0.449
Occupation	On the job	154(71.6)	129(72.5)	25(67.6)	0.363	0.547
	Unemployed	61(28.4)	49(27.5)	12(32.4)		
Education	≥ high school	138(64.2)	120(67.4)	18(48.6)	4.693	0.030^*^
	< high school	77(35.8)	58(32.6)	19(51.4)		
Current residence	Urban	207(96.3)	172(96.6)	35(94.6)	0.354	0.552
	Rural	8(3.7)	6(3.4)	2(5.4)		
Smoking	No	210(97.7)	173(97.2)	37(100)	1.064	0.302
	Yes	5(2.3)	5(2.8)	0		
Alcohol drinking	No	209(97.2)	173(97.2)	36(97.3)	0.001	0.972
	Yes	6(2.8)	5(2.8)	1(2.7)		
Sleep(h/d)		8.0(8.0,9.0)	8.0(7.5,9.0)	8.0(8.0,9.0)	-0.525	0.600
Exercise(MET) (minutes/w)		556.0 ± 911.3	597.7 ± 967.3	299.6 ± 343.4	1.326	0.187
**Clinical and obstetric data**						
Pre pregnancy BMI(kg/m^2^)		23.9 ± 4.0	23.3 ± 3.8	24.7 ± 5.1	-2.125	0.035^*^
History of chronic diseases	No	168(78.1)	138(77.5)	30(81.1)	0.226	0.634
	Yes	47(21.9)	40(22.5)	7(18.9)		
Family history of chronic diseases	No	163(75.8)	132(74.2)	31(83.8)	1.548	0.213
	Yes	52(24.2)	46(25.8)	6(16.2)		
Medicine use	No	181(84.2)	150(84.3)	31(83.8)	0.005	0.941
	Yes	34(15.8)	28(15.7)	6(16.2)		
Alanine aminotransferase (U/L)		16.2(11.8,23.0)	15.6(11.7,21.8)	16.5(11.1,22.5)	-0.054	0.957
Number of gravidities		2.0(1.0,2.0)	2.0(1.0,2.0)	1.5(1.0,2.5)	-1.898	0.058
Number of childbirths		0(0,1.0)	0(0,1.0)	0(0,1.0)	-1.211	0.226
Type of conception	Natural	198(92.1)	162(91.0)	36(97.3)	2.419	0.298
	Ovulation induction	6(2.8)	5(2.8)	1(2.7)		
	Artificial	11(5.1)	11(6.2)	0(0.0)		
Early pregnancy reaction	No	101(47.0)	87(48.9)	14(37.8)	1.499	0.221
	Yes	114(53.0)	91(51.1)	23(62.2)		
Depression(scores)		0(0,3.0)	1.0(0,3.0)	0(0,1.0)	-0.064	0.949
Anxiety(scores)		1.0(0,3.0)	1.0(0,3.0)	2.5(0,5.8)	-0.280	0.779

IFG: Impaired fasting blood glucose; BMI: Body mass index; ^*^: *P* < 0.05; T: Independent Samples t-test; χ2: Chi-square test; Z: Mann–Whitney U test

### The relationship between interoceptive sensitivity and fasting blood glucose in early pregnancy

As detailed in Table 3, logistic regression models were constructed to assess the association between IS (independent variable) and IFG (dependent variable), adjusting for covariates (educational level and pre-pregnancy BMI) identified from demographic and clinical data with *P* < 0.05. In Model 1, IS was a significant independent protective factor against IFG (OR = 0.429, 95% CI: 0.256, 0.721). After adjusting for pre-pregnancy BMI and educational level in both Model 2 and Model 3, IS remained significantly and inversely associated with IFG (OR = 0.491, 95%CI: 0.287, 0.839). In the fully adjusted model, neither educational nor pre-pregnancy BMI showed a significant association with IFG.

**Table 3:** Association between interoceptive sensitivity and impaired fasting glucose in early pregnancy

Variables	Model 1		Model 2		Model 3	
	*P*	OR (95%CI)	*P*	OR (95%CI)	*P*	OR (95%CI)
Interoceptive sensitivity	0.001^**^	0.429(0.256,0.721)	0.005^**^	0.467(0.274,0.797)	0.009^**^	0.491(0.287,0.839)
Education			0.183	1.664(0.786,3.524)	0.240	1.577(0.738,3.370)
Pre pregnancy BMI(kg/m^2^)					0.122	1.076(0.981,1.180)

Body mass index (BMI). ^**^: *P* < 0.01

### The status of dietary quality in early pregnancy

According to the BPI-P classification criteria [14], both DQD and food variety were categorized into different groups. DQD scores were categorized as follows: Q1 (0–12), representing relatively appropriate intake; while Q2 [13–24], Q3 (25–36) and Q4 (≥ 36) indicating low, moderate and high imbalance, respectively. Similarly, food variety was divided into three groups: Q1 (< -5.0), Q2 (-5.0 to -4.0), and Q3 (> -4.0).

As shown in Table 4, compared with the normal group, more pronounced dietary imbalance was shown in the IFG group. Specifically, the IFG group had higher overall DQD scores, with most participants classified as having moderate dietary imbalance. Further analysis revealed that the IFG group had significantly higher LBS than the normal group, a difference mainly driven by inadequate intake of dairy, soybeans and nuts (*P* = 0.024). In addition, the IFG group showed significantly lower food variety scores (*P* = 0.004), with 64.9% of participants in the lowest food variety quartile, which significantly higher than the 50.6% observed in the normal group.

**Table 4:** Comparison of healthy diet in normal group and IFG group with early pregnancy

Variables		Total (*N* = 215)	Normal group	IFG group	χ2/Z	*P*
		-	< 5.6 mmol/L (*N* = 178)	≥ 5.6 mmol/L (*N* = 37)	-	-
DQD (scores)	Q1(0 ~ 12)	1(0.5)	1(0.6)	0(0.0)	11.110	0.011^*^
	Q2(13 ~ 24)	76(35.3)	71(39.9)	5(13.5)		
	Q3(25 ~ 36)	121(56.3)	93(52.2)	28(75.7)		
	Q4(>36)	17(7.9)	13(7.3)	4(10.8)		
	LBS	22.0(18.0,26.0)	21.0(17.0,25.0)	24.0(21.0,28.0)	-2.626	0.009^**^
	HBS	4.0(0.0,6.0)	4.0(0.0,6.0)	4.0(1.0,5.0)	-0.199	0.842
DQD of Each variety	**1. Grains**	0(-2.0,2.0)	0.0(0.0,2.0)	0.0(-2.0,1.0)	-0.925	0.355
	**2. Vegetables and fruits**	-2.0(-5.0,-1.0)	-2.0(-5.0,0.0)	-3.0(-5.0,-1.0)	-0.687	0.492
	Vegetables	-2.0(-4.0,-1.0)	-2.0(-4.0,-1.0)	-3.0(-4.0,-1.0)	-1.558	0.119
	Fruits	0(-2.0,0.0)	0.0(-2.0,0.0)	0.0(-2.0,2.0)	-0.652	0.514
	**3. Dairy**,** soybeans and nuts**	-7.0(-12.0, -6.0)	-7.0(-11.0,-6.0)	-10.0(-12.0,-6.0)	-2.253	0.024^*^
	Soybeans and nuts	-6.0(-6.0,0.0)	-6.0(-6.0,0.0)	-6.0(-6.0,-4.0)	-1.357	0.175
	Dairy products	-6.0(-6.0,-1.0)	-6.0(-6.0,-1.0)	-6.0(-6.0,-1.0)	-1.455	0.146
	**4. Animal foods**	-4.0(-6.0,0.0)	-4.0(-7.0,0.0)	-3.0(-4.0,0)	-0.229	0.819
	**Poultry**	0.0(0.0,2.0)	0.0(0.0,2.0)	0.0(0.0.3.0)	-0.942	0.346
	Aquatic products	-4.0(-4.0,0.0)	-4.0(-4.0,0.0)	-4.0(-4.0,0.0)	-0.015	0.988
	Eggs	0(-4.0.0,2.0)	0.0(-4.0,2.0)	0.0(-4.0,1.0)	-0.473	0.636
Food variety	Q1(<-5.0)	114(53.1)	90(50.6)	24(64.9)	11.240	0.004^**^
	Q2(-5.0~ -4.0)	36(16.7)	26(14.6)	10(27.0)		
	Q3(>-4.0)	65(30.2)	62(34.8)	3(8.1)		

IFG: Impaired Fasting Glucose; LBS: Low Bound Score; HBS: High Bound Score; DQD: Diet Quality Distance; ^*^: *P* < 0.05; ^**^: *P* < 0.01; χ2: Chi-square test; Z: Mann–Whitney U test

### The Relationship between interoceptive sensitivity, diet quality, and impaired fasting glucose

#### Correlation analysis

Spearman correlation analysis revealed that IS showed a significant positive correlation with food variety (*r* = 0.169), but significant negative correlations with DQD (*r* = -0.228) and IFG (*r* = -0.224). IFG was positively correlated with DQD (*r* = 0.201) and negatively correlated with food variety (*r* = -0.163). Notably, a strong negative correlation was observed between DQD and food variety (*r* = -0.431). Detailed results are presented in Table 5.

**Table 5:** Correlation analysis of relationship of interoceptive sensitivity, diet quality and impaired fasting glucose

Variables		Interoceptive sensitivity	Diet quality		IFG
			DQD	Food variety	
Interoceptive sensitivity		-	-0.228^**^	0.169^*^	-0.224^**^
Diet quality	DQD	-0.228^**^	-	-0.431^***^	0.201^**^
	Food variety	0.169^*^	-0.431^***^	-	-0.163^*^
IFG		-0.224^**^	0.201^**^	-0.163^*^	-

#### Mediation effect model analysis

To explore the mediating roles of DQD and food variety in the relationship between IS and IFG, we constructed two separate mediation models. IS was treated as a continuous variable, and IFG as a binary outcome. In these models, IS served as the independent variable, DQD and food variety as the mediators (in Model 1 and Model 2, respectively), and IFG as the dependent variable. Because our proposed mediation model is fully saturated, it yields a perfect fit (CFI = 1.000, TLI = 1.000, RMSEA = 0.000, SRMR = 0.000). Therefore, our evaluation focuses exclusively on the estimated path coefficients.

**Fig. 1:**
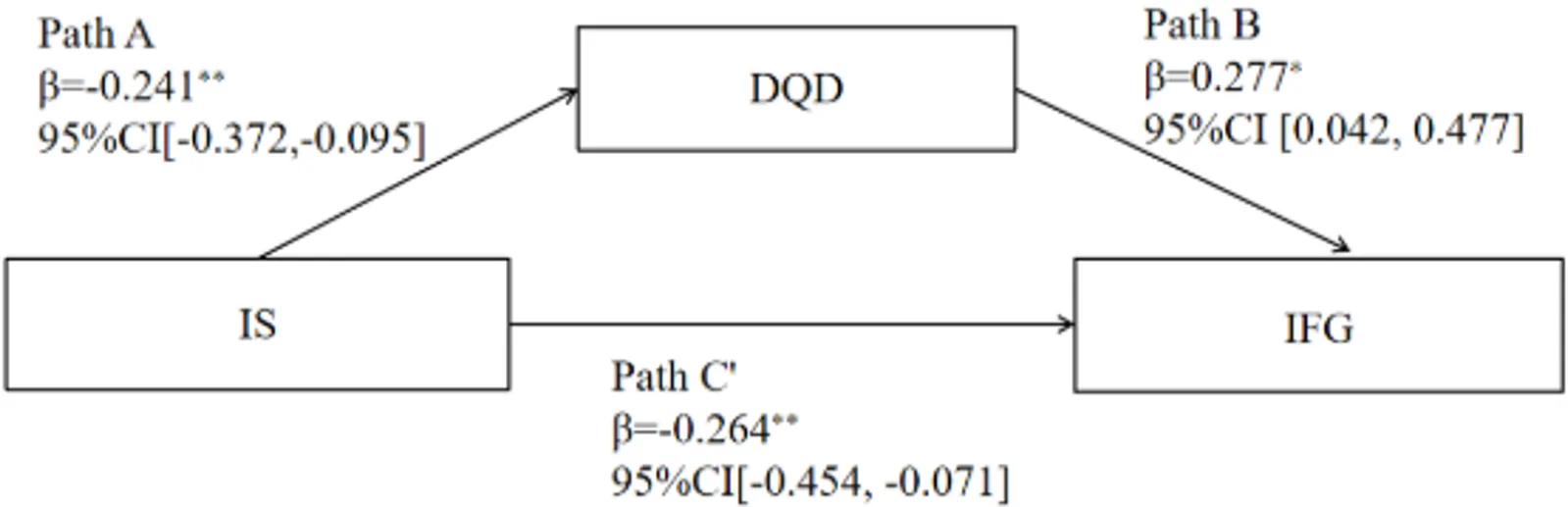
The Mediating Role of DQD between IS and IFG. Path A: IS → DQD; Path B: DQD → IFG; Path C’: IS → IFG, Direct Effect. *: *P* < 0.05; **: *P* < 0.01; Path coefficients shown in the model are standardized. Pre-pregnancy BMI and education level were controlled for as covariates in the mediation analysis

**Fig. 2:**
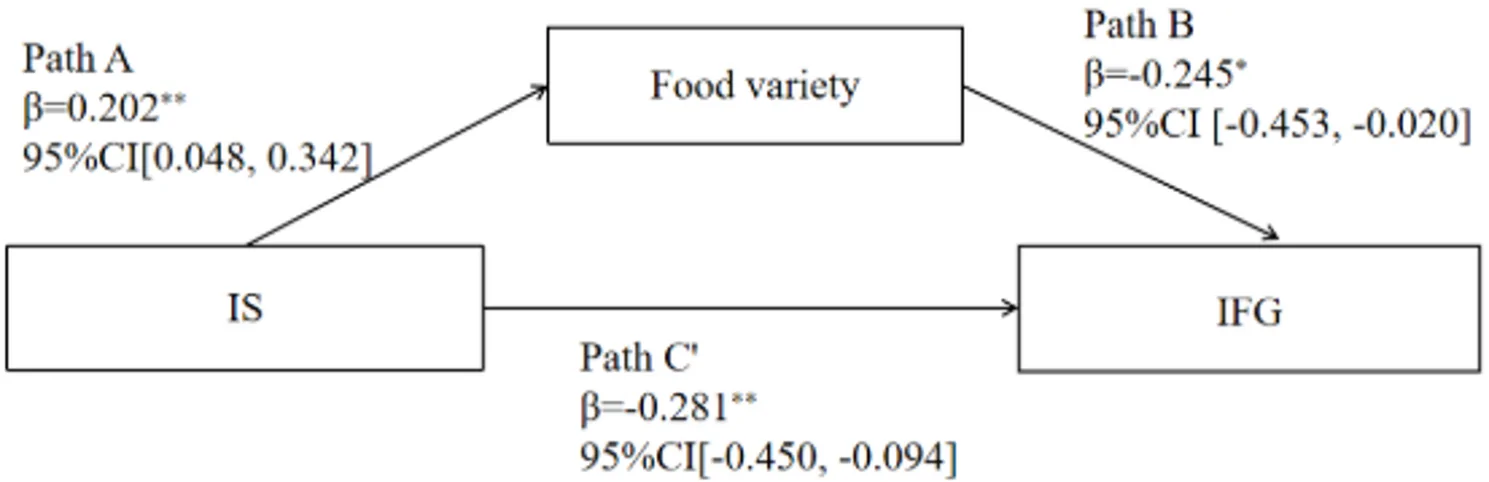
The Mediating Role of Food Variety between IS and IFG. Path A: IS → Food variety; Path B: Food variety → IFG; Path C’: IS → IFG, Direct Effect. *: *P* < 0.05; **: *P* < 0.01; Path coefficients shown in the model are standardized. Pre-pregnancy BMI and education level were controlled for as covariates in the mediation analysis

The path analysis for Model 1 (Fig. 1) indicated that IS was a significant negative predictor of both DQD (β = -0.241) and IFG (β = -0.264). Concurrently, DQD was significantly positively associated with IFG (β = 0.277). After controlling for the mediator, IS remained a significant negative predictor of IFG (β = -0.264). Mediation analysis (Table 6) confirmed that DQD (B = -0.101) played a partial mediating role in this relationship, accounting for 20.2% of the total effect. Similarly, Model 2 (Fig. 2) showed that IS significantly and positively predicted food variety (β = 0.202), while both IS (β = -0.281) and food variety (β = -0.245) negatively predicted IFG. Furthermore, food variety (B = -0.074) acted as a partial mediator between IS and IFG, accounting for 14.9% of the total effect.

**Table 6:** The mediating effect of dietary quality (DQD and food variety) between IS and IFG in early pregnancy

Mediating effect pathway		Effect size	95%CI
X-M1-Y	Direct effect	-0.400	(-0.719, -0.097)
	Indirect effect	-0.101	(-0.241, -0.018)
	Total effect	-0.501	(-0.806, -0.205)
X-M2-Y	Direct effect	-0.422	(-0.724, -0.131)
	Indirect effect	-0.074	(-0.207, -0.010)
	Total effect	-0.496	(-0.824, -0.202)

Notes: Unstandardized coefficients are reported. Impaired fasting glucose (IFG); X: interoceptive sensitivity; M1: DQD; Y: IFG; M2: food variety; ^*^: *P* < 0.05; ^**^: *P* < 0.01

## Discussion

In this study, we observed a significant association between interoceptive sensitivity and impaired fasting glucose in early pregnancy, which was mediated by dietary quality, mainly including diet quality distance and food variety.

### Prevalence of IFG in early pregnancy

The prevalence of IFG in this study was 17.2%, which aligns closely with the 17.6% reported by Wei et al. across 14 reproductive medicine centers in China [11].

### Status of interoceptive sensitivity in early pregnancy

At present, there were few studies reporting the statuses of interoceptive sensitivity in early pregnancy, especially for group with impaired fasting blood glucose. Crossland et al. found a mean interoceptive sensitivity score of 2.74 ± 0.34 among pregnant individuals [18]. Similarly, Noda et al. observed a mean interoceptive sensitivity score of 2.91 ± 0.40 in mid-to-late pregnancy [19]. In the present study, the overall median score for interoceptive sensitivity was 2.7 (2.0, 3.2), which is consistent with the findings of the above studies. In addition, the participants in this group had higher score on the *Not Distracting* dimension [3.5(2.3,4.3)] compared to the findings of Crossland et al. (2.2 ± 0.93) [18] which may be attributed to the different stages of pregnancy of participants, Crossland et al. included women across all three trimesters, our study focused on early pregnancy.

At same time, a distinct disparity was observed between groups: the normal glucose group had a median score of 2.7 (2.1, 3.3), whereas the IFG group scored lower at 2.1 (1.8, 2.7). Specifically, the IFG group demonstrated significantly reduced scores in the *Emotional Awareness* [2.4(0.6,3.2) vs. 3.0(2.0,3.8)], *Self-Regulation* [1.8(1.0,2.5) vs. 2.8(1.5,3.5)], *Body Listening* [1.3(0.3,3.0) vs. 2.3(1.0,3.0)], and *Trust* [2.3(1.0,3.0) vs. 3.0(2.0,4.0)] dimensions compared to the normal glucose group, indicating the interoceptive sensitivity of IFG group was lower than that of normal group.

### Statuses of dietary quality in early pregnancy

The main tools currently used to assess dietary quality include Dietary Diversity Score (DDS), Healthy Eating Index (HEI), Diet Quality Index-International (DQI-I), Dutch Healthy Diet (DHD) index, Dietary Balance Index for Pregnancy (DBI-P), and Sustainable Healthy Diets (SUSDIET), etc. Among those metrics, the DQI-I evaluates dietary quality across four dimensions: moderation, balance, variety, and adequacy. For example, Hayat et al. applied both the DQI-I and DDS to investigate dietary quality in pregnant women [20], the results indicated that the average scores of DQI-I and DDS among pregnant women were 60.46 ± 8.55 and 5.44 ± 1.70, respectively, suggesting that their dietary quality was generally at a relatively low level. The DBI-P was selected for this study largely due to its suitability for assessing the dietary patterns of the Chinese pregnant population.

In our study, the IFG group exhibited higher DQD scores, along with lower food variety scores, compared to the normal glucose group, indicating poorer overall dietary quality. This aligns with findings from a previous cohort study, which reported that diabetic patients had higher scores on the Dietary Quality Index-International compared to healthy subjects [21]. Furthermore, our observation of reduced food variety in the IFG group is supported by Liu et al., who identified a significant inverse association between dietary variety scores and the risk of new-onset diabetes (HR 0.85; 95% CI 0.80–0.90 per one-point increment) [22]. Regarding specific food categories, the IFG group in this study scored lower on dairy products, soybeans, and nuts. This is consistent with a meta-analysis of randomized controlled trials showing that low glycemic index/load (GI/GL) dietary patterns—characterized by the consumption of dairy, soybeans, and nuts—significantly reduced fasting glucose compared to control diets (mean difference = -0.36; 95% CI: -0.49, -0.23; *P* < 0.001) [23]. This study indicates that currently, pregnant women with impaired fasting glucose exhibit lower dietary quality, primarily reflected in relatively low intake of dairy products, soybeans, and nuts, as well as insufficient dietary diversity.

### Association between interoceptive sensitivity and impaired fasting glucose in early pregnancy

To the best of our knowledge, few studies have explored the relationship between interoceptive sensitivity and impaired fasting glucose during early pregnancy. Our finding suggests that interoceptive sensitivity is significantly associated with the incidence of impaired fasting glucose (OR = 0.491; 95% CI: 0.287, 0.839). The possible mechanism is that blunted interoceptive sensitivity may impair the detection of glucose signals, therefore impact appetite-regulation and glucose metabolism. Consistent with this, Young et al. observed that greater interoceptive accuracy correlated with lower blood glucose level [24], highlighting the regulatory function of interoception in glycemic control.

### Mediating effect of dietary quality on the association between interoceptive sensitivity and fasting glucose

Our mediation analysis revealed a significant indirect pathway wherein dietary quality mediates the association between interoceptive sensitivity and fasting glucose levels.

Firstly, few studies have directly investigated the relationship between interoceptive sensitivity and dietary quality. Our results bridge this gap by showing that interoceptive sensitivity correlates with DQD and food variety. We proposed that lower interoceptive sensitivity may be associated with difficulties in perceiving internal hunger and satiety cues [25], leading to a disconnection from physiological needs. This regulatory failure was likely manifested by the higher dietary imbalance (high DQD), mainly insufficiency intake (high LBS). This may relate to an insufficient intake of low glycemic index foods such as nuts and dairy products, which can destabilize blood glucose levels; on the other hand, the reduced food diversity seen in these participants limits the intake of nutrients, subsequently impacting glucose metabolism. This aligns with findings from a 1-year postpartum study, which reported that intuitive eating skills during pregnancy predict better future diet quality. In particular, the ability to rely on hunger and satiety cues was significantly associated with higher diet quality scores (β = 2.03; 95% CI: 0.09, 3.97) and increased adherence to dairy and fiber intake recommendations [26]. Meghana et al. [27] demonstrated that higher diet quality, as measured by the HEI-2010 score, was associated with better glycemic outcomes. Similarly, Hosseini et al. [28] reported that participants in the top quartile of healthy food diversity scores had a significantly lower risk of developing high fasting blood glucose (HR 0.11; 95% CI: 0.02, 0.21; *P* = 0.004) compared to those in the lowest quartile. These findings are consistent with our observation that lower dietary quality and variety are linked to impaired fasting glucose.

Thus, this study supported our research hypothesis that IS is associated with impaired fasting blood glucose. This relationship may be mediated by dietary quality in early pregnancy. Our findings suggest a pathway where lower interoceptive sensitivity may relate to a reliance on less optimal eating patterns, characterized by high LBS and insufficient intake of food variety. Although the absolute indirect effects are modest, DQD and food variety account for 20.2% and 14.9% of the total effect, respectively. This insufficient intake of food variety and healthier foods leading to IFG may be attributed to overall blood glucose fluctuations or decreased insulin sensitivity in early pregnancy, highlighting the practical significance of modifying dietary behaviors for glycemic regulation. Importantly, dietary quality and variety during pregnancy are shaped not only by internal sensory signals but also within a broader behavioral and psychosocial context. Recent research indicates that dietary patterns are closely linked to nutrition knowledge, physical activity, and dieting self-efficacy [29]. In this light, higher interoceptive sensibility may interact with these cognitive and behavioral factors—such as boosting a pregnant woman’s dieting self-efficacy and behavioral control—to facilitate healthier, more diverse dietary choices, which subsequently support optimal glycemic outcomes. Therefore, clinical attention should be directed toward supporting healthy, balanced dietary patterns in early pregnancy to help maintain optimal glycemic stability, rather than relying solely on post-onset interventions.

### Limitations

This study has several limitations. First, the cross-sectional design precludes causal inference regarding the impact of interoception on glucose metabolism, and the temporal relationship among interoceptive sensitivity, dietary quality, and IFG cannot be determined. The mediation analysis should be interpreted as an exploratory statistical pathway rather than definitive evidence of a causal mediating mechanism. Second, our assessment of interoceptive sensitivity relied on the self-reported MAIA-2, which measures subjective interoceptive sensibility rather than objective interoceptive accuracy. Similarly, while the three-day dietary record is a validated method, it may not fully capture long-term habitual dietary intake, and our analysis did not account for other relevant nutritional variables such as total energy intake, carbohydrate intake, glycemic index/load, or meal timing. Third, although our IFG prevalence of 17.2% is highly representative of the national average (17.6%), the small absolute size of this group (*N* = 37) may limit the statistical power and stability of our logistic regression and mediation models, and the regional, early-pregnancy sample may limit the generalizability of our findings to other regions or later gestational stages. Future studies should employ randomized, population-based sampling across larger multicenter networks to confirm these findings in a broader demographic. Despite these limitations, our study provides novel insights into the behavioral pathways linking body awareness to metabolic health.

## Conclusion

Our study demonstrates that lower interoceptive sensitivity is closely linked to impaired fasting glucose in early pregnancy, a relationship partially mediated by dietary quality. Specifically, lower interoceptive sensibility may be associated with less effective intuitive dietary regulation, which is linked to greater dietary imbalance and higher fasting glucose levels. These findings offer a novel perspective on the potential value of interoception-oriented approaches during early gestation. Incorporating body-awareness training into early pregnancy care may represent a promising avenue to help support healthy dietary choices and optimize glycemic outcomes for pregnant women at risk of metabolic dysfunction.

## Data Availability

The datasets used and/or analyzed during the current study are available from the corresponding author upon reasonable request.

## Abbreviations

IS: Interoceptive sensitivity
IFG: Impaired fasting glucose
FPG: Fasting plasma glucose
DBI-P: Diet Balance Index for Pregnancy
DQD: Dietary quality distance
LBS: Low Bound Score
HBS: High Bound Score
GDM: Gestational diabetes mellitus
CNS: Central nervous system
IPAQ-SF: International Physical Activity Questionnaire–Short Form
GAD-7: Generalized Anxiety Disorder 7-item
PHQ-9: Patient Health Questionnaire-9
BMI: Body mass index
MAIA-2: Multidimensional Assessment of Interoceptive Awareness–Version 2
